# What the American Medical Association Asks of the State Societies

**Published:** 1902-03

**Authors:** 


### What the American Medical Association Asks of the State Societies.

[I reproduce the following, from the *Journal of the American Medical Association,* that members of the Texas State Medical Association may know, and when we meet in Dallas, May 6th, be prepared to vote intelligently on the changes that are contemplated to be made in the constitution of the State Medical Association; also, that members of local societies may be informed of the new relations that will be brought about if the changes above mentioned are effected. It is most desirable that there should be a full attendance. It is also highly important that those who are not members of any society should catch on, quick, because things are shaping now, so that to be not a member of one's local society puts one *hors du concours;* that is, not in the swim, the push, not even a little bit. A badge of membership will be the *sine qua non* of respectability, in fact.—ED.]

The following resolutions, among others, recommended by the committee on reorganization were adopted at the last meeting of the American Medical Association:

*c.* That the State societies unitedly agree to federate themselves in the American Medical Association, and, as a preliminary to this, adopt a uniform organic law in regard to certain fundamental principles, viz.: to divide their annual sessions into two branches, legislative and scientific; the legislative branch to be as small as is compatible with representation from all the county societies, and to be composed of delegates elected by the county societies.

*d.* That membership in the county or district societies shall constitute membership in the respective State society without further dues, and that no one be admitted to membership in the State society except through county or regular district societies.

*e.* That funds to meet the expenses of the State society be raised by a per capita assessment on the county and district societies.

*f.* That a united effort be made to influence special societies to limit their membership to those who support the regular organization, and the semi-national and miscellaneous societies to encourage systematic organization, by covering a definite territory and also by limiting their membership to supporters of the regular organization.

*g.* That each State society create a permanent committee and a fund for the purpose of enforcing all medical laws in every part of its territory.

*h.* That each State society co-operate with the American Medical Association and with the other State societies in solving the problems now before the profession relating to medical education, medical legislation, reciprocity, licensing, etc.

An analysis of these resolutions shows that the American Medical Association requests the following:

1. The federation of all the State associations in the American Medical Association.

2. That all associations adopt a uniform plan of organization as regards certain fundamental principles.

3. That each State association have two distinct branches, legislative and scientific.

4. That the legislative branch be as small as compatible with representation from all county societies in the State or territory, and to be composed of delegates elected by the county (or district) societies.

5. That the scientific branch be composed of and open to all members of county (or district) societies, or as stated in the resolution: "Membership in the county or district society shall constitute membership in the respective State societies without further dues, and that no one be admitted to membership in the State society except through county or regular district societies."—*Journal American Medical Association.*

